# Supplementary material for: DMRforPairs: identifying Differentially Methylated Regions between unique samples using array based methylation profiles
Source: BMC Bioinformatics. 2014 May 15;15:141. doi: 10.1186/1471-2105-15-141 (PMC4046028; doi:10.1186/1471-2105-15-141)
Supplement: Additional file 2 — DMRforPairs output for the comparison of A431-MCF7 and NA17018-NA17105. Please start from the HTML files in each folder. Available via the BMC Bioinformatics website. [file 1471-2105-15-141-S2.zip › 1394847754114233_MOESM2_ESM/A431_MCF7/figures/7065.pdf]

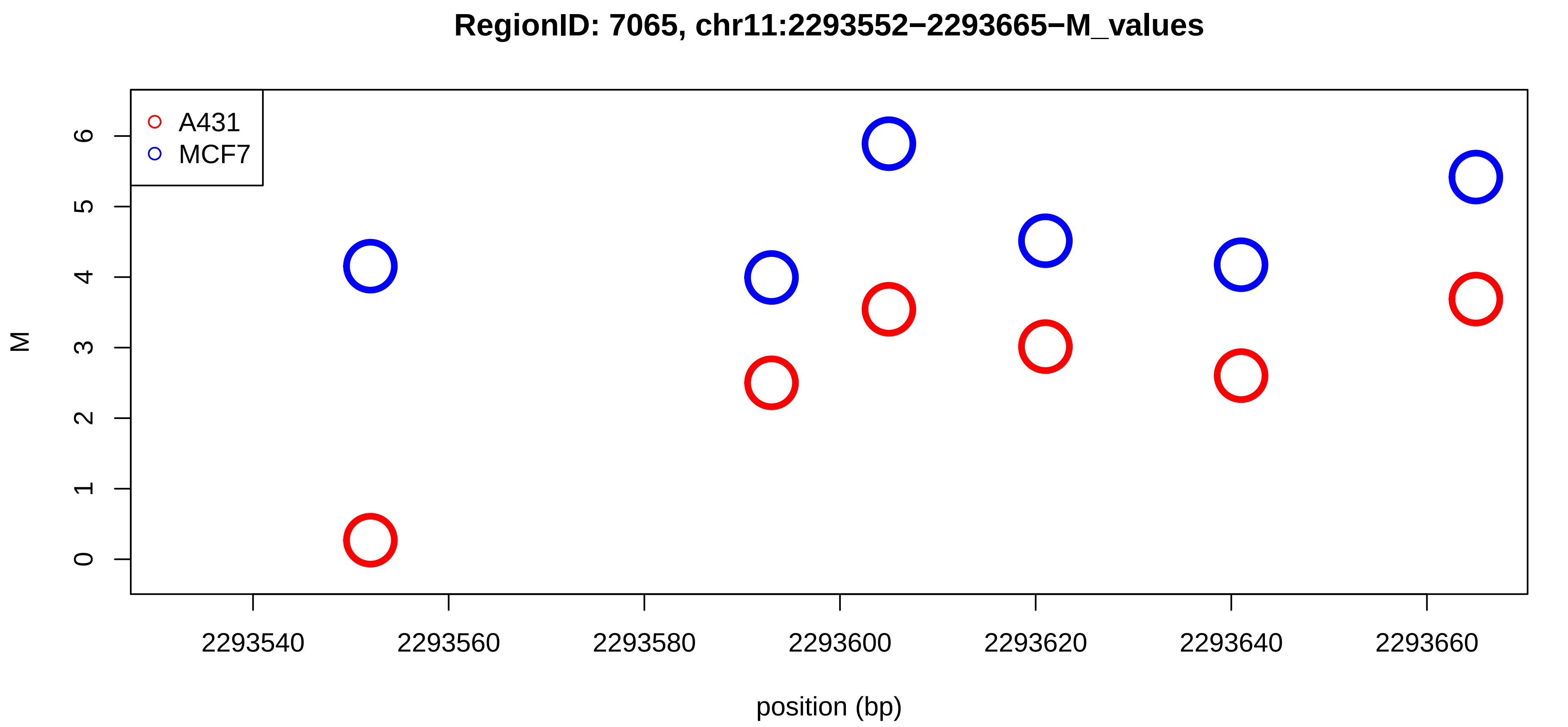

RegionID: 7065, chr11:2293552−2293665−M_values
A431
MCF7
M
0
1
2
3
4
5
6
2293540
2293560
2293580
2293600
2293620
2293640
2293660
position (bp)

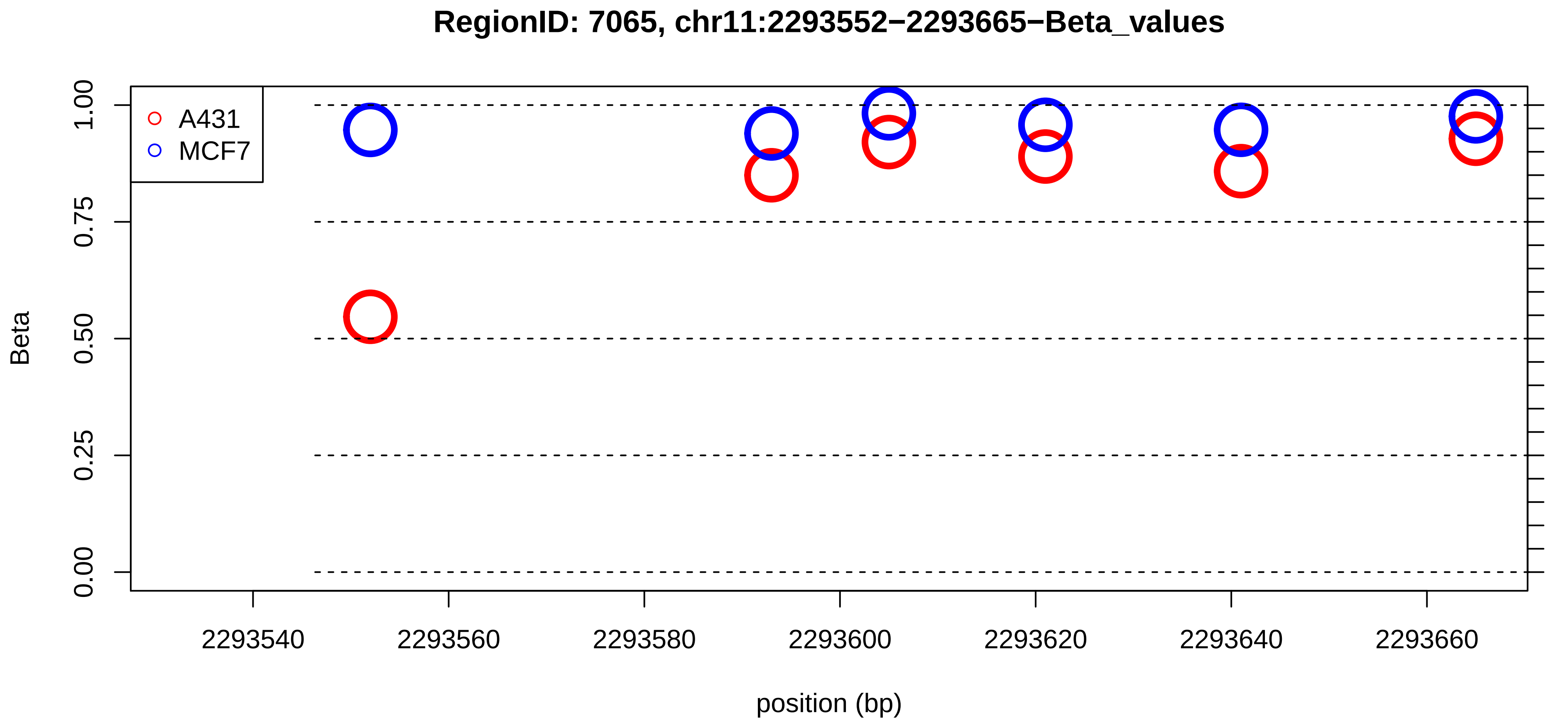

RegionID: 7065, chr11:2293552−2293665−Beta_values
A431
MCF7
Beta
0.00
0.25
0.50
0.75
1.00
2293540
2293560
2293580
2293600
2293620
2293640
2293660
position (bp)

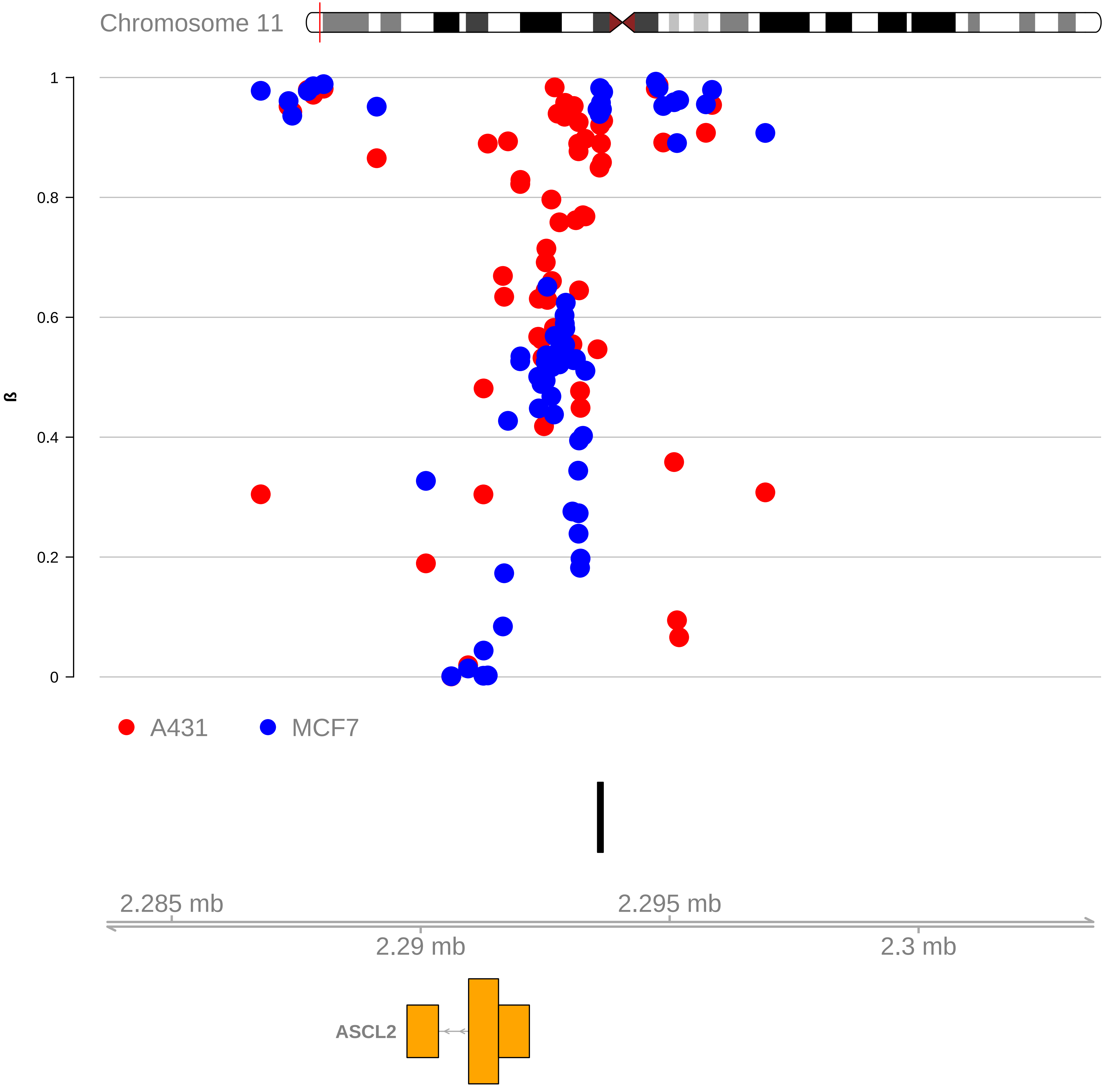

Chromosome 11
1
0.8
0.6
0.4
0.2
0
β
A431
MCF7
2.285 mb
2.29 mb
2.295 mb
2.3 mb
ASCL2
